# Association between pulse pressure and markers of cognitive function: a systematic review and meta-analysis

**DOI:** 10.1097/HJH.0000000000004401

**Published:** 2026-07-24

**Authors:** Olivia Gaunt, Kaavya Venkatesh, Ryan McNally, Phil J. Chowienczyk, Marina Cecelja

**Affiliations:** a King's College London, Faculty of Life Sciences and Medicine, Sport and Exercise Medical Sciences; b King's College London, Faculty of Life Sciences and Medicine, GKT School of Medical Education; c King's College London, King's Health Partners Centre for Translational Medicine; d King's College London British Heart Foundation, Cardiovascular Division, Department of Clinical Pharmacology, St Thomas’ Hospital; e King's College London, Department of Twin Research, Ageing and Genomic Epidemiology, London, UK

**Keywords:** attention, cognition, cognitive decline, cognitive function, executive function, language, memory, processing speed, pulse pressure, visuospatial ability

## Abstract

Our aim was to systematically review and meta-analyse evidence on the association between pulse pressure (PP) and cognitive function using PubMed, PsycInfo, Embase and Scopus (inception–July 2025) publication databases. Studies were included if they reported an association between PP and cognitive function and summarized narratively and by performing fixed-effects meta-analysis. The search identified 4171 publications with 43 studies meeting inclusion criteria. Domains assessed included global cognition, memory, language, attention, executive function, processing speed and visuospatial ability. Meta-analysis suggests a positive association between PP and global cognition, and a negative association with memory in both cross-sectional and longitudinal studies with inconsistent findings from narratively summarized studies. Processing speed, executive function and language negatively associated with PP in cross-sectional studies with limited evidence provided by longitudinal studies or narratively summarized studies. There was limited evidence of an association with attention and visuospatial ability.

## INTRODUCTION

The co-existence of two or more chronic diseases is now characteristic of a worldwide ageing population and an overwhelming public health concern [1]. Cardiovascular disease contributes significantly to morbidity and mortality with over 16% of individuals over the age of 60 living with cardiovascular disease in the UK [2]. It is increasingly recognized that the presence of cardiovascular disease may also account for other age-related comorbidities. Cognitive decline is of particular importance with mild cognitive impairment affecting between 5 and 20% of people over 65, and one in six of these progressing to dementia within 1 year [3]. Dementia risk increases with the presence of cardiovascular disease, with multiple cardiovascular conditions increasing the risk of dementia by more than five times [4]. Whilst the association of cognitive decline and cardiovascular disease may occur through age being a common risk factor, other shared risk factors and a direct link between vascular disease and vascular damage in the brain are now thought to contribute to this association [5].

Arterial stiffening is a ubiquitous characteristic of vascular ageing and is highly predictive of major adverse cardiovascular events [6]. It is thought to increase blood pressure pulsatility, and consequent damage to small arteries through raised pulse pressure, particularly in highly vascular organs such as the brain, may contribute to subsequent cognitive decline [7]. Several studies have associated PP to measures of cognitive function with variable findings. A previous systematic review investigating the association between PP and cognitive function did not distinguish between different domains [8]. Understanding mechanisms underlying the association between cardiovascular disease (CVD) and cognitive decline, and the possible role of PP, would inform on potential preventive strategies. The aim of the present study was to perform a systematic review and meta-analysis of publications investigating the association between PP and various cognitive function domains (Table 1).

**TABLE 1:** Summary of studies investigation the association between pulse pressure and cognitive function tests in chronological order

Ref.	Participants	Cognitive function test	Measured PP	Association	Extra notes
Robert *et al.*[40].	1379 participants from UK Biobank, age range 44–82 (median 65, 52% women)	G-score of five cognitive tests: Pairs Memory (standardized and reversed), Reaction Time (standardized and reversed), Prospective Memory Fluid Intelligence (IQ), and Numeric Memory	Peripheral PP (OMRON)	PP was associated with performances on all cognitive tests in univariate Model 1, and after adjusting for all covariates, PP remained negatively associated with performances on Numeric Memory (*ß* = −0.028, *P* = 0.002), Fluid IQ (*ß* = −0.060, *P* < 0.001), and *g*score (*ß* = −0.018, *P* < 0.001), independent of age, sex, ethnicity, education and vascular risk factors.	Covariates include age, sex, ethnicity, education, smoking status and frequent alcohol use.
Parada *et al.*[51]	146 community-dwelling older adults free of dementia (mean age 77 years, 62% women)	Cognitive function assessed using Trails B.	Longitudinal measurements of brachial PP trajectories over a mean follow-up of 24 years using a mercury sphygmomanometer.	PP trajectories did not associate with Trails B (beta = -7.61, *P* = 0.024), but higher PP trajectory was associated with poorer Trails B performance via reduced restricted isotropic diffusion.	Adjusted for age, sex, education, alcohol consumption, smoking, exercise, BMI, history of CV disease and history of diabetes.
Li *et al.*[34]	190 hospitalized patients with Alzheimer's disease (mean age 62 years, 57% men)	Cognitive function was assessed using AVLT, AFT and SDMT.	24-h ambulatory PP (MOBIL-O-GRAPH).	There was no significant association between cognitive function tests to ambulatory PP.	Covariates included age and course of disease.
Lamar *et al.*[33]	167 Black adults without dementia (mean age 76 years, 86% women) recruited from the Minority Aging Research Study.	Cognitive function was assessed using 19 tests, including episodic memory, semantic memory, working memory, perceptual speed and visuospatial ability (measured longitudinally over 4.5 years).	Peripheral PP (OMRON)	PP was not associated with baseline or change in global cognition but did associated negatively with change in episodic memory (*P* = 0.042) but not episodic or semantic memory. PP and WMH interaction associated baseline working memory.	Covariates included age, sex, years of education, BMI and diabetes.
Qin *et al.*[39]	89 healthy older adults (mean age 69 years, 66% women)	Working memory capacity (WMC)	Peripheral PP	PP was a significant predictor of WMC in older adults (β = −0.27, *P* < 0.05).	Controlled for age, sex and education
Mizuhara *et al.*[16]	2546 Japanese participants free of dementia (mean age 61 years, 54% men)	Cognitive function was assessed using Okabe's test and Kohs’ tests.	Brachial PP	Higher PP associated with lower Okabe (*P* = 0.002) and Kohs’ scores (*P* = 0.019).	Covariates included age, sex and medical history of hypertension, diabetes mellitus and dyslipidaemia.
Gyanwali *et al.*[28]	373 memory clinic patients, (mean age 72.1 ± 7.7 (55.2% women)	MMSE and MoCA.	Brachial PP (Omron)	Mean 24hr PP association with MMSE; OR = 1.00, *P* = 0.979, with MoCA; OR = 1.04, *P* = 0.647	Adjusted for age, sex, use of antihypertensives, smoking status, heart conditions, diabetes, hyperlipidaemia, stroke history, dementia diagnosis, presence of brain vascular lesion and CDR score.
Hu *et al.*[11]	1546 nondemented participants (mean age 62 years, 40% women) from the Chinese Alzheimer's Biomarker and Lifestyle study	China-modified MMSE, Montreal Cognitive Assessment Score (MoCA).	Peripheral PP (Omron)	Higher PP associated with decreased cognitive function in total participants (MoCA, β= − 0.1022, *P* < 0.001). Results were similar in mid-life (MoCA, β= − 0.0840, *P* < 0.05) and late-life groups (MoCA, β= − 0.1186, *P* < 0.05). Results were similar after further adjustment. The association between PP and cognitive impairment was partially mediated by tau pathologies, with with mediation varying from 15 to 24% (*P* < 0.05).	Adjusted for age, sex, years of education and APOEε4 status. Further adjustment included BMI, cigarette use, alcohol use, medical history, status of antihypertensive treatment, coefficient of variation in cerebrospinal fluid biomarker measurements.
Moonen *et al.*[36]	2498 participants from the Age, Gene/Environment Susceptibility (AGES)–Reykjavik Study (mean age 80 years, 41% men)	Cognitive function measures included verbal memory (California Verbal Learning Test), working memory (Digits backwards, Cambridge Neuropsychological Test Automated Battery spatial working memory test, Stroop Test Part III), processing speed (Digit Symbol Substitution Test, Figure Comparison, Stroop Test Part I and II).	Peripheral PP	PP associated with working memory (beta = -0.002, *P* = 0.023) but not processing speed (*P* = 0.151) or verbal memory scores (*P* = 0.0096).	Adjusted for sex, education, brain volume, coronary calcium, BMI, DM, heart failure, smoking, stroke, midlife BP, use of antihypertensive medication.
Caballero *et al.*[52].	781 participants were volunteer community-dwelling adults from the Victoria Longitudinal Study (mean age 71 years, 67% female).	Executive function was measured by measures for inhibition (Hayling, Stroop), updating (Computational Span, Reading Span) and for shifting (Brixton, Color Trails, Letter Series, Letter Sets).	PP	Lower PP associated with lower executive function decline.	Longitudinal analysis was used to measure executive function trajectories.
Zang *et al.*[15].	3009 participants from the SPRINT-MIND study with 755 participants undergoing MRI (mean age 69 years, 37% women).	Cognitive tests of five cognition domains, including global cognition, executive function, attention, memory and language.	PP	PP negatively associated with global cognition (β = −0.048, *P* = 0.008), executive function (β = −0.014, *P* = 0.040), attention (β = −0.013, *P* = 0.035), memory (β = −0.021, *P* = 0.045), and language (β = −0.020, *P* = 0.001).	Covariates included age, sex, race, education, smoking, drinking, BMI, cardiovascular disease, cholesterol, glucose, eGFR, medication. Mediation analysis showed that increased WML volume contributed to 10.8% of global cognition, 9.5% of executive function, 10.6% of memory, and 7.2% of language decline associated with PP.
Shi *et al.*[41]	1375 participants from the Alzheimer's Disease Neuroimaging initiative (age 73 years, 55% men).	ADNI-MEM (composite *z* score based on item-level data from the Rey Auditory Verbal Learning Test, the Mini-Mental State Examination (MMSE), the AD Assessment Scale Cognitive Test, and Logical Memory I and II) and EDNI-EF (item-level data from the Trail Making Test Parts A and B, Digit Span Backward, Digit Symbol, Animal Fluency, Vegetable Fluency, and Clock Drawing Test) neuropsychological tests.	Peripheral PP	In cross-sectional analysis PP did not associate with memory or executive function. Longitudinally, baseline high PP associated worse memory performance (beta = -0.018, *P* = 0.058).	Adjusted for age, gender, education APOE ε4 carrier status, vascular risk factors (BMI, T2DM, hyperlipidaemia, hypertension, CV disease), cognitive diagnosis and intracranial volume.
Levine *et al.*[12]	432 dementia-free individuals ≥45 (median age, 66; 45% women) with stroke (92% ischemic); from the BASIC project.	MMSE (3MSE = modified version), AFT and Trail Making Tests A and B.	Peripheral PP (Omron) 90 days after stroke.	Significant increase in association per 10mmHg increase for 3MSE: −0.06 (−0.11, −0.01) *P* = 0.01. For AFT −0.14 (−0.31, − 0.04) *P* = 0.13. For Trails A −0.02 (−0.03, −0.01) *P* = 0.005. For Trails B −0.01 (−0.02, −0.006) *P* < 0.001.	Age, education, race/ethnicity, BMI, diabetes, depressive symptoms, antihypertensive medication.
Snitz *et al.*[47]	100 participants of the Ginkgo Evaluation of Memory Study (mean age 92 years, 38% women)	Cognitive tests included CVLT, Rey-Osterrieth figure delayed recall, semantic fluency and TRAIL B. Mean follow-up was 12 years.	Peripheral PP	Higher PP predicted longitudinal executive function (Trails B, beta = 0.01, *P* = 0.008) and language test (semantic fluency; beta = -0.06, *P* = 0.024) but not verbal memory test (CVLT delayed recall), visual memory test (Rey-Osterrieth figure delayed recall).	No adjustment for covariates.
Le DuBose *et al.*[48]	113 middle-aged/older adults (mean age 67 years, 50% women)	Cognitive tests include RBANS and Stroop Colour Word Reading Test.	Central PP (Sphygmocor)	cPP negatively associated with processing speed (beta = -0.01, *P* < 0.05), executive function (beta = -0.22, *P* < 0.05)	Adjusted for age, sex and antihypertensive use. There was a significant interaction between cPP and education which moderated the association with processing speed but not executive function.
Lipnicki *et al.*[17]	Meta-analysis from 20 population-based longitudinal cohorts including 48 522 individuals (mean age 73 years, 58% women) aged 54–105 (mean = 72.7) without dementia at baseline. Studies had 2–15 years of follow-up.	Global cognitive composite outcome (memory, language, processing speed and executive functioning tests) and MMSE.	Peripheral PP	No significant association with PP was found with the global cognitive composite or MMSE cross-sectionally or with cognitive decline.	Covariates include age, education and sex.
Lamballais *et al.*[20]	5187 adults from Rotterdam Study. Mean age 61.8 (42.9% male)	Stroop task, letter digit substitution test, verbal fluency test, delayed recall score of 15-word learning test, Purdue pegboard test. G-factor then calculated from test scores.	Peripheral PP	PP (β = −0.044, 95% CI = [−0.069; −0.020]) showed statistically significant negative associations with the g-factor.	Further adjusted for education level, BMI, smoking status, diabetes status, use of BP meds, diet quality score, alcohol intake and physical activity standardized per cohort.
Werhane *et al.*[27]	738 normally ageing participants from the Alzheimer's Disease Neuroimaging Initiative that were followed up over 4 years (mean age at baseline 73 years, 48% female).	Functional Activities Questionnaire (FAQ).	Peripheral PP (mercury Sphygmomanometer)	There was no significant association between baseline PP and FAQ score. Elevated PP predicted greater functional difficulty trajectories across four years of follow-up (beta = 0.313, *P* < 0.001).	Adjusted for age, education, sex, depressive symptoms and antihypertensive medication.
Montoya *et al.*[24]	72 HIV+ and 36 HIV- community-dwelling, older (i.e. aged 50 and above) adults who participated in the California HIV/AIDS Research Program Successfully Aging Seniors with HIV study at the UCSD HIV Neurobehavioral Research Program (mean age 58.5 years, 76.9% men)	Participants completed a comprehensive neurocognitive test battery (administration time: 2–2.5 h) that assesses seven neurocognitive domains consistent with Frascati recommendations for neuroAIDS research [30]. The seven neurocognitive domains were speed of information processing (SIP), learning, memory, executive functioning, verbal fluency, working memory, and fine motor functioning.	Brachial PP (sphygmomanometer)	The best model (model 3) identified that both the linear (β = .41, *P* < .01) and quadratic (β = −.33, *P* = .01) terms for PP were statistically significant in the model for neurocognitive function.	Model 3 adjusted for HIV status, diabetes, VEGF, Tie-2, and the HIV interactions with VEGF and Tie-2, in addition to testing both linear and quadratic pulse pressure effects on cognition.
Li *et al.*[21]	108 CVSD patients aged 45--80	MoCA	24-h ambulatory PP	Correlation analysis of 24hour mean PP and MoCA score; r = 0.367, *P* = 0.031	
Tsao *et al.*[46]	1223 Framingham Offspring Cohort participants (mean age 62 years, 56% women) with follow-up at 6 years.	Neurocognitive testing (Trails Parts B and A and Similarities tests).	Central PP (tonometry)	Higher baseline CPP associated with greater progression of neurocognitive decline as measured by Similarities test (beta = -0.08 ± 0.03, *P* = 0.013) but not Trails B-A.	Covariates include age, sex, time between visits, diabetes, atrial fibrillation, smoking, antihypertensive therapy, cardiovascular disease, APOE genotype and obesity.
Riba-Llena *et al.*[18]	699 participants from the Investigating Silent Strokes in Hypertensive Individuals Study (mean age 63 years, 50% women).	Cognitive performance was determined using a battery of cognitive tests evaluating memory, attention, processing speed, executive function, motor and visuospatial functions.	Office and 24-h ambulatory PP	Daytime PP negatively associated with attention (beta = -0.22) but not other cognitive parameters after adjustment. Nocturnal PP associated with mild cognitive impairment (OR per s.d. increase in nocturnal PP = 2.254, 95% CI 1.029, 4.939).	Covariates included age, sex, education, antihypertensive medication, antiplatelet and statin treatments. Results were similar after further adjustment for cerebral small vessel disease.
Nation *et al.*[45]	454 stroke and dementia free Framingham Offspring Cohort study participants (mean age 60 year, 53% women).	Cognitive measures included measures of verbal memory (Wechsler Memory Scale), delayed free recall, visual memory, attention and executive functioning (Halstead–Reitan Trail Making Test Part A [Trails A] and Part B [Trails B]), visuospatial organization or nonmotor executive visuoconstructional skills (Hooper Visual Organization Test) and language (Boston Naming Test) over a 5–7 year follow-up.	Brachial PP	Baseline PP associated with worse executive ability, and longitudinal decline in executive ability.	Covariates included age, sex, education, assessment interval and interim stroke.
Lefferts *et al.*[49].	2058 adults from the 1999–2002 NHANES (mean age 70 years, 55% women).	Digit-symbol substitution test (DSST)	Peripheral PP.	PP was significantly associated with DSST (β = -0.07, *P* < 0.05).	Covariates included age, sex, race, education, BMI, smoking, diabetes, chronic disease, hypertensive and dyslipidaemia medication.
Wang *et al.*[30].	289 stroke/TIA hospital patients. Mean age 74.8 ± 9.6 (56.2% men, 43.8% women)	MMSE and CDR	Peripheral PP	A U-shaped association was also found between PP and cognitive decline indexed by MMSE. Adjusted odds ratio's over quartiles: 2.7, 2.0, 3.3. *P*-values 0.34, 0.110, 0.005.	Adjusted for age, education, baseline CDR score, MTLA, ARWMC total score, recurrent stroke, DBP, AF or CHF, admission NIHSS score and antihypertensive therapy on discharge.
McFall *et al.*[44].	570 older adults from the Victoria Longitudinal study longitudinal follow-up over 9 years (mean age 71 years, 65% women).	Episodic (word recall, Rey auditory verbal learning) and semantic memory (fast recall and vocabulary)	PP	Baseline PP predicted episodic memory performance at age 75 (b = -.125, *P* < .05) and rate of 9-year EM (b = -.008, *P* < .001) but not semantic memory.	
Ogliari *et al.*[14]	1,540 outpatients from the hospital-based Milan Geriatrics 75+ Cohort Study (mean age 82 years, 70% women).	MMSE	PP.	Higher PP associated with higher MMSE score (*P* < 0.05).	Covariates included age, sex, education, medication, renal function.
Pase *et al.*[38]	493 independently living adults (mean age 53 years, 61% women).	Cognitive performance assessed using Swinburne University Computerised Cognitive Assessment Battery (SUCCAB) reflecting processing speed, Stroop processing, spatial working memory and recognition memory.	Central (Sphygmocor) and brachial (Omron) PP	Higher central PP associated with poorer processing speed (*P* < 0.05), Stroop processing (*P* < 0.001), and recognition memory (*P* < 0.01) but not spatial working memory. Higher brachial PP associated with poorer Stroop processing (*P* < 0.01) but not processing speed, spatial working and recognition memory.	Covariates included age, sex, education, height, weight, heart rate, MAP, antihypertensive and cholesterol lowering medication.
Tsao *et al.*[42]	1,587 stroke and dementia-free Framingham Offspring Study participants (mean age 61 years, 45% men).	Cognition was assessed using Trails B minus Trails A and logical memory delayed recall.	Central PP	Central PP associated with worse logical memory, delayed recall memory (*P* < 0.05) but not Trail B - Trail A.	Covariates included diabetes, atrial fibrillation, ECG left ventricular hypertrophy, smoking, antihypertensive medication, cardiovascular disease, central obesity, education, depression, total and high density lipoprotein, triglycerides and homocystein.
Yaneva-Sirakova *et al.*[25]	148 hypertensive participants (mean age 64 years, 35% men)	MMSE and MoCA.	Ambulatory PP	MMSE and MoCA was significantly different (negatively) between participants with elevated PP (*P* < 0.05).	
Sabayan *et al.*[26]	572 community-dwelling individuals followed-up for 5 years (67% women).	MMSE	PP	Higher PP (*P* = .008) at age 85 was associated with lower annual declines in MMSE score. There was no significant cross-sectional association.	Covariates included sex, education, smoking, alcohol, history of stroke, antihypertensive medication, diabetes, history of cardiovascular disease.
Chrysohoou *et al.*[10]	535 participants from the Ikaria Island (mean age 76 years, 53% men)	MMSE	Brachial PP	High PP associated with lower MMSE score (odds ratio 0.45, 05% CI: 0.22–0.92).	Age, sex, smoking, cardiovascular disease, creatinine clearance, hypertension, diabetes, obesity, physical activity and education.
Benetos *et al.*[32].	873 participants from the PARTAGE cohort (mean age 87 years, 79% women)	MMSE	PP	No association was found between PP and MMSE.	
Raz *et al.*[50]	158 healthy adults (mean age 52 years)	Executive functions -Wisconsin cart sorting test, 3 back task, size judgement span task.	Brachial PP (Omron)	No significant association between PP and executive function was found.	Covariates included age, sex and hypertension related polymorphisms
Mitchell *et al.*[35]	668 participants from the community-based Age, Gene/Environment Susceptibility – Reykjavik study (mean age 75 years, 57% women).	Cognitive scores assessed using a battery of tests.	Carotid PP	cPP associated with lower memory scores (*P* = 0.013) and executive function but not speed.	Covariates include age, height, weight, heart rate, mean arterial pressure, high-density lipoprotein cholesterol, glucose, hypertension treatment and statin use.
Nation *et al.*[22]	109 participants (mean age 74 years, 44% men).	Neurological and neuropsychological evaluations (DRS, Wechsler Adult Intelligence Scale, Boston Naming Test, Wisconsin Card Sorting Test, Trail Making Test Part B, California Verbal Learning Test).	Peripheral PP	PP inversely correlated with language ability (*β* = −0.26; *P* < .01), but showed no significant relationship with other cognitive domains (global, attention, visuospatial, executive, memory, processing speed) after correction for multiple comparisons.	Adjusted for age, education and atrial fibrillation.
Pase *et al.*[37]	92 healthy individuals (mean age 53 years, 64% women).	Computerised cognitive tests consisting of immediate word recall, simple reaction time, digit vigilance, choice reaction time, tracking, spatial working memory, numeric working memory, delayed word recall, delayed word recognition and delayed picture recognition.	Central PP (Sphygmocor)	PP associated with episodic secondary memory (beta = -0.27, *P* < 0.05) and speed of memory retrieval (beta = 0.24, *P* < 0.05). Working memory, power of attention and continuity of attention did not associate with pulse pressure.	Covariates included were MAP, education, age, sex and BMI.
Molander *et al.*[31]	102 individuals aged >85 years followed-up over 5 years (mean age 87 years, 76% women).	MMSE.	PP	PP did not associate with decline in MMSE.	Adjusted for age and sex.
Dahle *et al.*[43]	104 healthy adults (mean age 54 years).	Cognitive performance -	PP	Elevated PP associated with slower processing speed (beta = -0.31, *P* < 0.01) but not executive functions, working memory, declarative memory.	
Triantafyllidi *et al.*[23]	110 nondiabetic patients aged 40–80 years (mean age 53.8 ± 11.2 years, 57 men) with recently diagnosed stage I–II essential hypertension.	MMSE	Brachial PP (both office and 24 h)	In the entire population, MMSE was negatively correlated with 24-h pulse pressure (PP) (r = −0.18, *P* < 0.05)	
Waldstein *et al.*[29]	1749 participants from the Baltimore Longitudinal Study of Aging followed up over 14 years (mean age 57 years, 53% men).	Tests of verbal and nonverbal memory, attention, perceptuo-motor speed, confrontation naming, executive functions, and cognitive screening measures	PP (Sphygmomanometer)	Increasing levels of PP associated with a prospective decline on tests of verbal learning, nonverbal memory, working memory, and cognitive screening measures (*P* < 0.05).	Age, sex, BMI, education, depression score, cholesterol, smoking, alcohol, antihypertensive medication.
Obisesan *et al.*[13].	6163 participants from the Third National Health and Nutrition Examination Survey (mean age 71 years, 57% women).	MMSE	Brachial PP (sphygmomanometer)	PP associated with better cognitive performance as measured by MMSE in participants aged 60–69 (*P* = 0.02). In participants aged 70–79 PP was negatively associated with MMSE (*P* < 0.001). There was a nonlinear association in participants aged over 80 years.	Adjusted for age, sex, ethnicity, education, income, history of stroke, BMI, glycosylated haemoglobin, physical activity.
Robbins *et al.*[19].	Caucasian--American and African--American participants in the Maine-Syracuse Longitudinal Study (mean age 49 years, 57% female). 1563 participants of whom 147 were African-American.	Wechsler Adult Intelligence Scale subtests (Global Composite, Information, Similarities, Digit Span Forward, Digit Span Backward, Digit Symbol Substitution, and Block Design).	PP	Pulse pressure was inversely related to each cognitive variable, with the exception of Digit Span Backward.	Covariates included age, education, sex, occupation, race, antihypertensive medication, alcohol, smoking, psychotropic medication, diabetes, BMI, anxiety and depressive score.

## MATERIALS AND METHODS

### Data sources and searches

Pubmed, PsycInfo, Embase and Scopus were searched for published literature. PubMed comprises more than 34 million citations for biomedical literature from MEDLINE, life science journals, and online books. Key words for the search were “brain ageing” combined with “pulse pressure”. This included MeSH terms: brain; “brain”[MeSH Terms] OR “brain”[All Fields] OR “brains”[All Fields] OR “brain's”[All Fields]. Ageing: “aging”[MeSH Terms] OR “aging”[All Fields] OR “ageing”[All Fields]. Pulse pressure: “blood pressure”[MeSH Terms] OR (“blood”[All Fields] AND “pressure”[All Fields]) OR “blood pressure”[All Fields] OR (“pulse”[All Fields] AND “pressure”[All Fields]) OR “pulse pressure”[All Fields] from inception to 09.07.2025. In addition, a manual search of citation lists of relevant publications was also searched.

### Selection criteria

The list of titles and abstracts were initially screened for relevancy from the PubMed search. Articles were rejected if they did not investigate the association between PP and cognitive function; did not measure PP; were a review article; were a protocol paper/cohort profile or conference abstract; were animal studies; were a guideline/opinion paper; were a study in children; and were a case report. Relevant articles were retrieved and read in full. This was restricted to the English language. Articles that fulfilled the following criteria were included in the final review measured PP (articles where SBP), DBP and mean arterial BP were measured but not PP were excluded); investigated the association between PP and measures of cognitive function. No restriction was applied for inclusion of confounders. The reference list of all included articles was additionally checked for relevant studies and included if they fulfilled the above criteria.

### Data extraction

A single standardized form was used for data extraction, which included first author, publication year, sample size, participant characteristics (including percentage of men and women), method of measuring cognitive function, method pulse pressure was measured, association detected and confounders included in the model and other relevant information.

### Meta-regression analysis

To perform the meta-analysis, measures of association between PP and cognitive function were included. Outcome association measures collected included beta coefficient, odds ratio, 95% confidence intervals (95% CI), standard error and/or *P*-value. Publications not reporting appropriate outcome measures were narratively summarized. If standard error was not available, it was calculated from the *P*-value, 95% CI and beta coefficient. For longitudinal studies, the association for baseline PP and change in outcome measure was reported and trajectories of PP were narratively summarized. For the meta-analysis, a pooled effect size and 95% CI were calculated using the *metan* function in STATA. Results were displayed graphically using a forest plot. Association measures adjusted for the highest number of covariates were considered in the first instance. Finally, when studies reported comparison of mean values by groups without regression models or correlation coefficients, these were narratively summarised. Heterogeneity was assessed using *I*^2^ statistics and by evaluating the range of the pooled effect size [9]. Publication bias was evaluated using Egger's regression asymmetry test and visual funnel plot inspection.

## RESULTS

**FIGURE 1:**
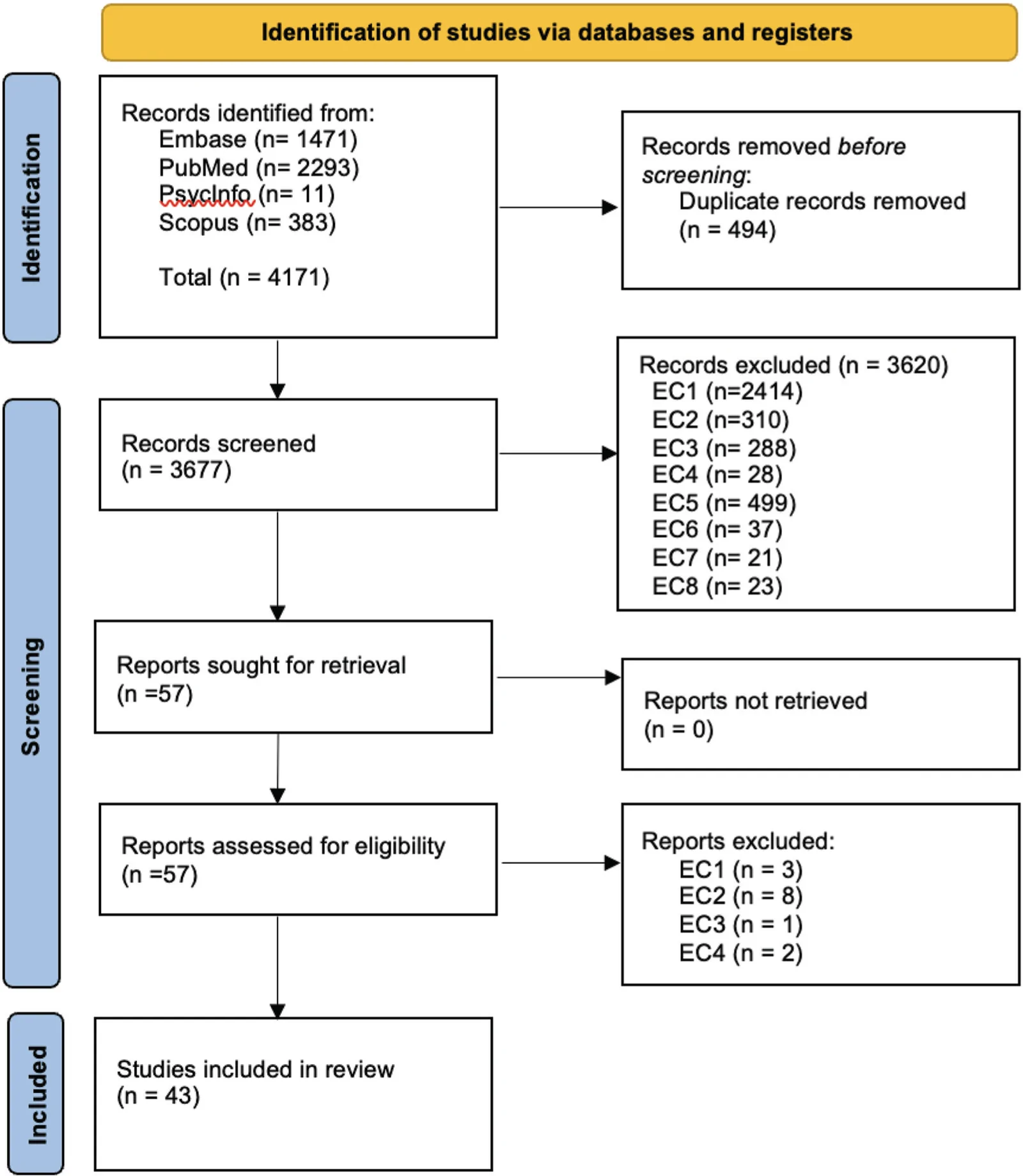
PRISMA flow chart of included and excluded publications [68].

The initial literature search identified 4171 publications. The publication title and abstract were initially checked for relevancy and of these, 3620 were excluded based on the exclusion criteria (Fig. 1). References of all 57 included studies were checked for relevancy and a further 14 publications excluded in line with the criteria, therefore 43 studies remained. Table 1 outlines studies investigating the association between PP and cognitive function tests.

### Global cognition

**FIGURE 2:**
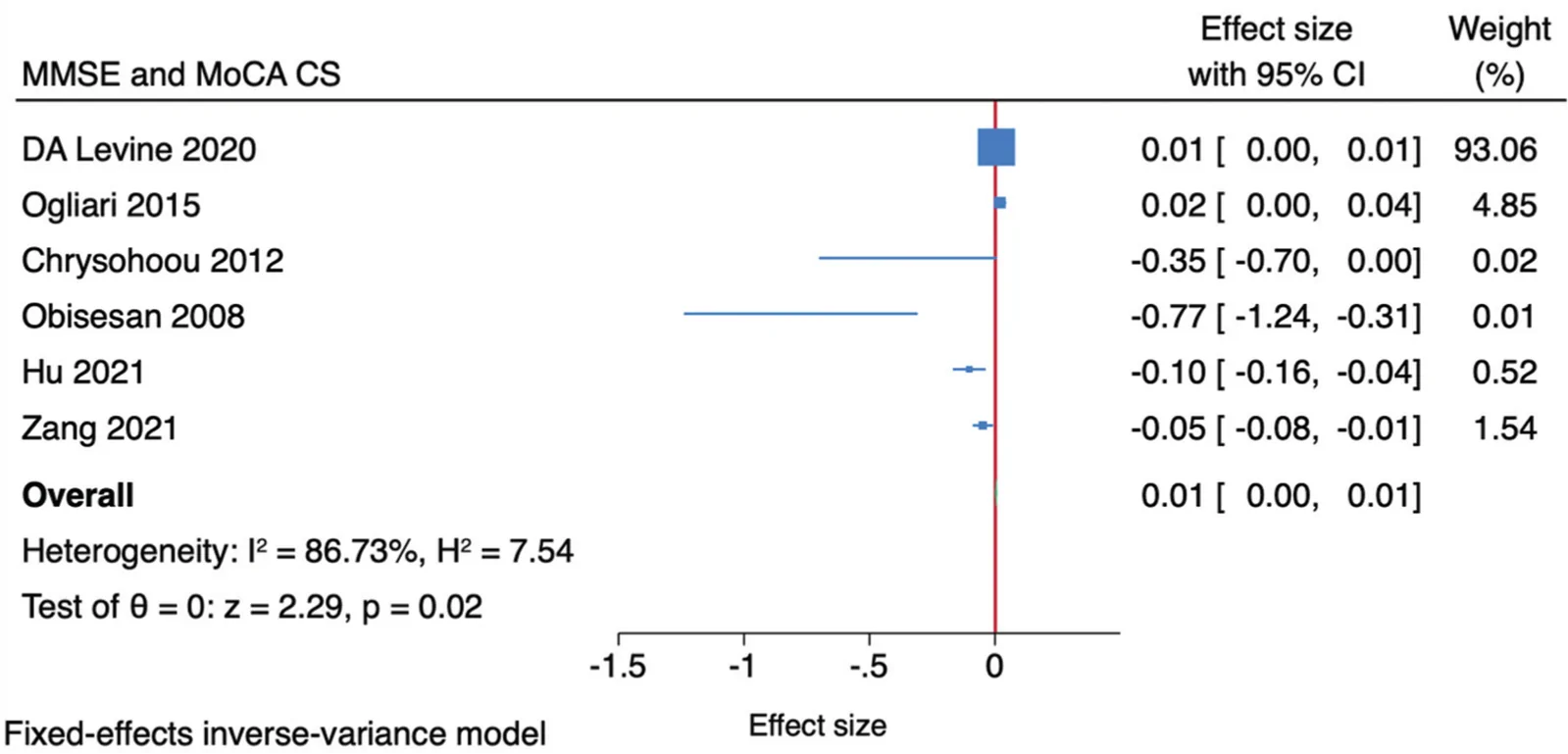
Meta-analysis results for the cross-sectional association between pulse pressure and global cognition.

Nineteen studies involving 73 635 participants assessed the cross-sectional association between PP and global cognition (Supplement Table 1). Of these, seven used MMSE and five used MoCA with the remaining using a combination of other cognitive function test (including DRS, Okabe's test, Koh's test, Wechsler Adult Intelligence Scale subtests, battery composite and functional activity questionnaires, Table 2). Meta-analysis of six studies [10–15] reporting standardised beta-coefficients or odds ratio (converted to standardised beta-coefficients) using MMSE or MoCA to assess global cognition. This gave a pooled standardized beta-coefficient of 0.005 (95% CI: 0.001–0.010, *P* = 0.0218, Fig. 2) with evidence of heterogeneity between studies indicated by a wide range of standardized beta regression coefficient (-0.77 to 0.02 and *I*^2^ = 86.73%, *P* < 0.001). Publication bias is suggested from funnel plot asymmetry and Egger's test (*P* < 0.001). Sensitivity analysis including only studies assessing global cognition using MMSE increased the pooled standardised regression coefficient minimally to 0.007 (95% CI: 0.002–0.011, *P* = 0.0038). Studies not included in the meta-analysis reported inconsistent findings, with one study (*n* = 2546) showing a significant negative association [16] when global cognition was assessed using Okabe's and Koh's test; three studies (*n* = 50 784) did not report any statistical findings [17–19]; four studies found a significant negative association [20–23], whilst two studies showed a positive association when global cognition was assessed using a comprehensive neurocognitive test battery and MMSE [24,25]. In a group comparison study of *N* = 572 participants, there was modest evidence that MMSE score changed across tertiles of PP (*P* = 0.06) [26]. The remaining study found no significant association when assessing the association between PP and global cognition when using a functional activity questionnaire [27].

**TABLE 2:** Summary of cognitive tests

Phenotype	Brief description of cognitive test	Cognition phenotype measures
Halstead-Reitan Trail Making Test Part B (Trails B)	Consists of 24 circles on a piece of paper with half the circles containing the numbers 1–12 and the other half containing the letters A-L. Participants are asked to draw lines connecting the circles in ascending order, alternating between connecting numbers and letters with a maximum completion time of 300 s. Higher completion times indicate poorer performance.	A test of visuomotor tracking and executive function.
The Repeatable Battery for the Assessment of Neuropsychological Status (RBANS)	Compromises 12 subtests that takes 30 min to administer.	Immediate memory, visuospatial/constructional perception, attention, language and delayed memory.
Mini-Mental State Examination (MMSE)	Involves asking a series of questions with a maximum score of 30 points. A score below 24 indicates moderate cognitive impairment.	Tests orientation, registration, attention, calculation and recall.
Digits Backward	Participants are asked to repeat a series of digits backwards. List increases with each level succeeded.	Assesses working memory.
CANTAB spatial working memory test	A number of coloured squares are shown on a screen. By touching the boxes and using a process of elimination the participant will find one blue ’token’ in each of a number of boxes used to fill an empty column.	Spatial working memory.
Stroop Test	Participants are required to read 3 different tables as quickly as possible. Two of them represent condition in which participants are required to read names of colours printed in black ink and name different colour patches. The third table, colour-words are printed in an inconsistent colour (e.g. the word “red” is printed in green ink) and participants are required to name the colour instead of reading the word.	Assesses executive function.
Auditory Verbal Learning Test (AVLT); also Rey auditory verbal learning.	Five presentations of a 15-word list are given followed by attempted recall, including immediate recall, short delayed recall and long-delayed recall. The procedure is carried out a total of 5 times.	Measure ability to encode, combine, store and recover verbal information at different stages of immediate memory.
Animal Fluency Test (AFT)	The participant is asked to name as many animals as possible within a 60-s timeframe. A lower score represents poorer language function. Naming 15 or fewer animals may indicate cognitive impairment.	Assess language and executive function.
Symbol Digit Modalities Test (SDMT)	In 90 s participants are asked to pair specific numbers with given geometric figures. A lower score indicates worse attention.	Assess attention and processing speed.
Digit symbol substitution test (DSST)	Participants are asked to draw symbols paired with numbers [1–9] according to a key. Number of correctly matched symbols within 120 s was summed or the score (max score of 133).	Executive function
Montreal Cognitive Assessment Score (MoCA)	Compilation of neuropsychological tests that aim to assess executive function and attention. MoCA includes also the Trial-making test B variant, cub-drawing, clock-drawing, naming, memory, forward digit span, backward digit span, vigilance, attention – the serial 7-s test, sentence repetition, language fluency, abstraction, late recall, orientation. With a maximum score of 30 points, a score below 26 indicates moderate cognitive impairment.	Executive function and attention.
Similarities test	A greater number of similarities discerned reflects a better score.	Index of abstract reasoning reflecting executive function.
The Koh's block design test (Kohs’ test)	Participants are showed cards with a variety of coloured designs and are asked to reproduce them using a set of coloured blocks which is quantified using a intelligence quotient.	Assesses visuospatial ability in addition to executive function.
Working memory capacity (WMC)	Computation span task - participants are presented with a series of math problems and asked to indicate if it is correct. Participants then recall second number of the equation. Numbers of equations increased after every three trials with a total of 7 tasks. Reading span task - In this task, participants saw a sentence on the screen and indicated whether the sentence was true or false. Participants then typed in the last word of the sentence. The number of sentences increased by one every three trials, with a total of seven possible tasks.	Memory system with limited capacity that can be used for cognitive tasks.
Functional Activities Questionnaire (FAQ)	Consists of 10 items that assesses the participants level of performance on daily activities, including (1) writing checks, paying bills or balancing a check book; (2) assembling tax records; (3) shopping alone for clothes; (4) plying a game of skill such as chess; (5) heating water; (6) preparing a balanced meal; (7) keeping track of current events; (8) paying attention to and understanding a TV programme; (9) remembering appointments; (10) travelling out of the neighbourhood. Score ranges from 0 to 30 with a higher score rating higher dependence.	Measures activities of daily living. May be used to differentiate those with mild cognitive impairment and mild Alzheimer's disease.
Wechsler Adult Intelligence Scale	Tests of Information (information measures retention of general information), Similarities (similarities measures verbal concept formation and general mental ability), Digit Span Forward (measures attention and immediate memory span), Digit Span Backward (measures attention, immediate memory span, and ability to double track), Digit Symbol Substitution (measures attention, visual-motor coordination, and response speed), and Block Design (measures spatial perception and organization).	Assess a wide variety of cognitive abilities.
Okabe's Intelligence Scale (Okabe's test)	Modified Wechsler Adult Intelligence Scale-Revised for the Japanese population with a test score total of 60 points	Includes orientation, semantic memory, calculation, forward and backward digit span and paired association memory.
Wisconsin Card Sorting Test	Participants match a card with one of the four key cards according to the colour, shape, or number of geometric designs on the target card with category switch occurring after 10 successive correct trials. The participants, who were informed whether each response was correct or incorrect, continued until achieving six categories or completing 128 trials. The indices of performance were the total number of errors.	Executive function.
California Verbal Learning Test (CVLT) (ref Kaplan, 1991)	Involves immediate recall, short delay free recall, short delay cued recall, long delay free recall, long delay cued recall, long delay recognition.	Measures episodic verbal learning and memory.
Swinburne University Computerised Cognitive Assessment Battery (SUCCAB)	Involves simple reaction time, reaction time, immediate and delayed recognition memory, stroop congruent, stroop incongruent, spatial working memory, contextual recognition memory.	Reaction time, episodic memory, working memory, reaction time, attention.
Verbal Fluency Test (VFT)	Involves assessing an individual's ability to generate words from a specified letter of the alphabet or a semantic category.	Efficiency of searching in long-term memory.
Purdue Pegboard Test	Involves placing as many pins in parallel rows of holes using left and right hand simultaneously in 30 s.	Dexterity and fine motor skill.
Delayed Recall 15-word Test	Involves delayed recall of words (0–15 words) 10 min after visual presentation.	Retrieval from verbal memory.
UKB Reaction time (standardised and reversed)	Participants are presented with two cards with a symbol on them. In this Go/No-Go test, they were instructed that when the cards matched, they had to push a button. When the cards were different, they were to do nothing until a new pair of cards appeared. The score was their mean reaction time, in milliseconds, to press the button.	Processing speed
UKB Numeric memory	A backwards digit span task aimed to assess working memory. Participants were shown a list of numbers and instructed to recall the digit sequence in a reverse order. The score (0–12) was the maximum number of digits correctly remembered in reverse order.	Working memory
UKB Fluid IQ	Participants were presented with 13 multiple-choice questions. The score was the number of questions answered correctly in two minutes.	Verbal and numerical reasoning
UKB Prospective memory test	Participants are instructed to touch the Orange Circle at the end of the tests. At the end of the cognitive test battery, an instruction to touch the Blue Square (conflicting instructions) was presented to the participants. The score was coded as 2 if they correctly touched the Orange Circle on their first attempt, 1 if they correctly answered on their second attempt, and 0 if they incorrectly touched any other shape.	Prospective memory
UKB Pairs Matching (log(x + 1) transformed, and reversed)	Participants are presented on a screen with pairs of matching cards arranged randomly. The cards were then turned face down and participants were instructed to touch the cards to match up all the pairs of cards in the fewest number of touches. The score was the number of errors made before matching all pairs.	Visual declarative memory

**FIGURE 3:**
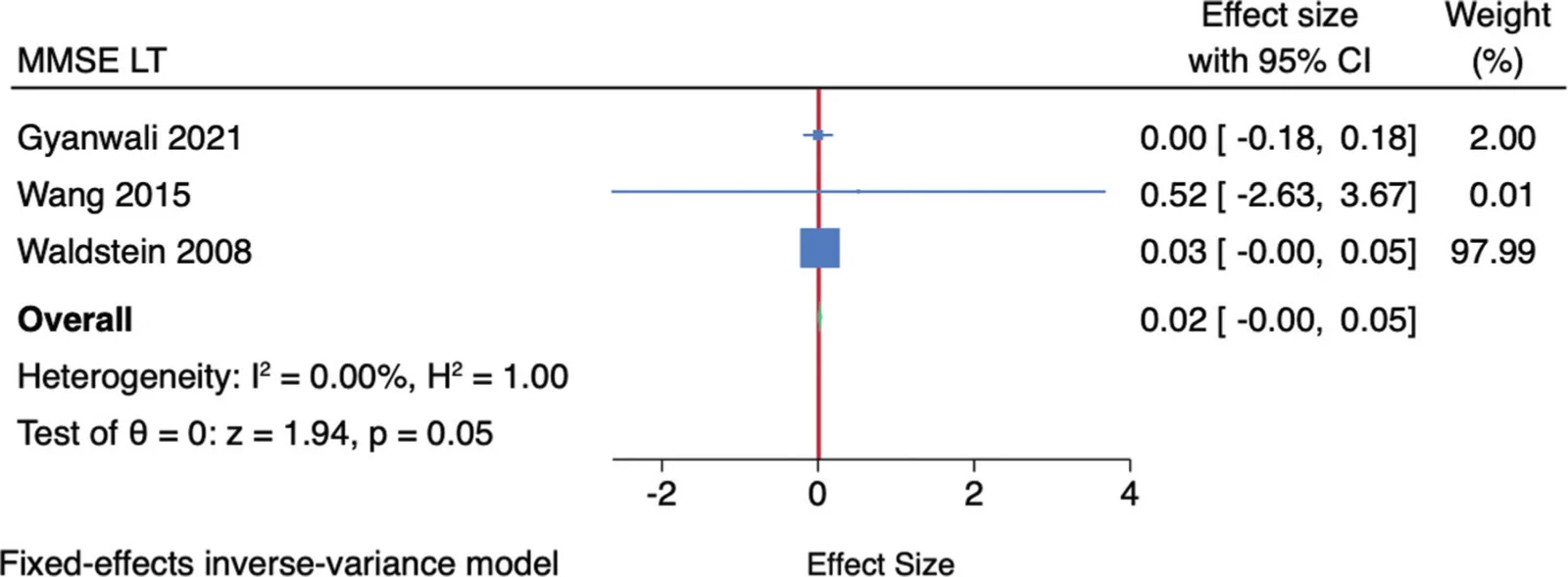
Meta-analysis results for longitudinal association of pulse pressure with global cognition.

Eight studies assessed the longitudinal association between PP and global cognition, involving 52 416 participants (Supplementary Table 1). Of these, three assessed MMSE and one study also assessed MoCA with the remaining assessing global cognition using battery composite and functional activity questionnaire. Meta-analysis of three studies [28–30] reporting standardized beta coefficients or odds ratio (converted to standardized beta coefficients, Supplementary Table 3) using MMSE to assess global cognition. This gave a pooled standardized beta coefficient of 0.025 (95% CI, -0.001 to 0.049, *P* = 0.0512, Fig. 3) with no evidence of heterogeneity between studies indicated by minimal range of standardized beta regression coefficient; (0.001 - 0.518 and *I*^2^ = 0.00%, *P* = 0.918). Publication bias was not suggested from funnel plot asymmetry and Egger's test (*P* = 0.958). Studies not included in the meta-analysis did not report any statistical association with one [28] using MoCA and another using MMSE [31] to assess the association between PP and global cognition longitudinally. Two studies *N* = 48 522 and *N* = 873 did not report any statistical association [17,32]. A group comparison study of *N* = 572 assessed over an average of 3 years found higher tertiles of PP were associated with lower annual decline in MMSE score (*P* = 0.008) [26].

### Memory

**FIGURE 4:**
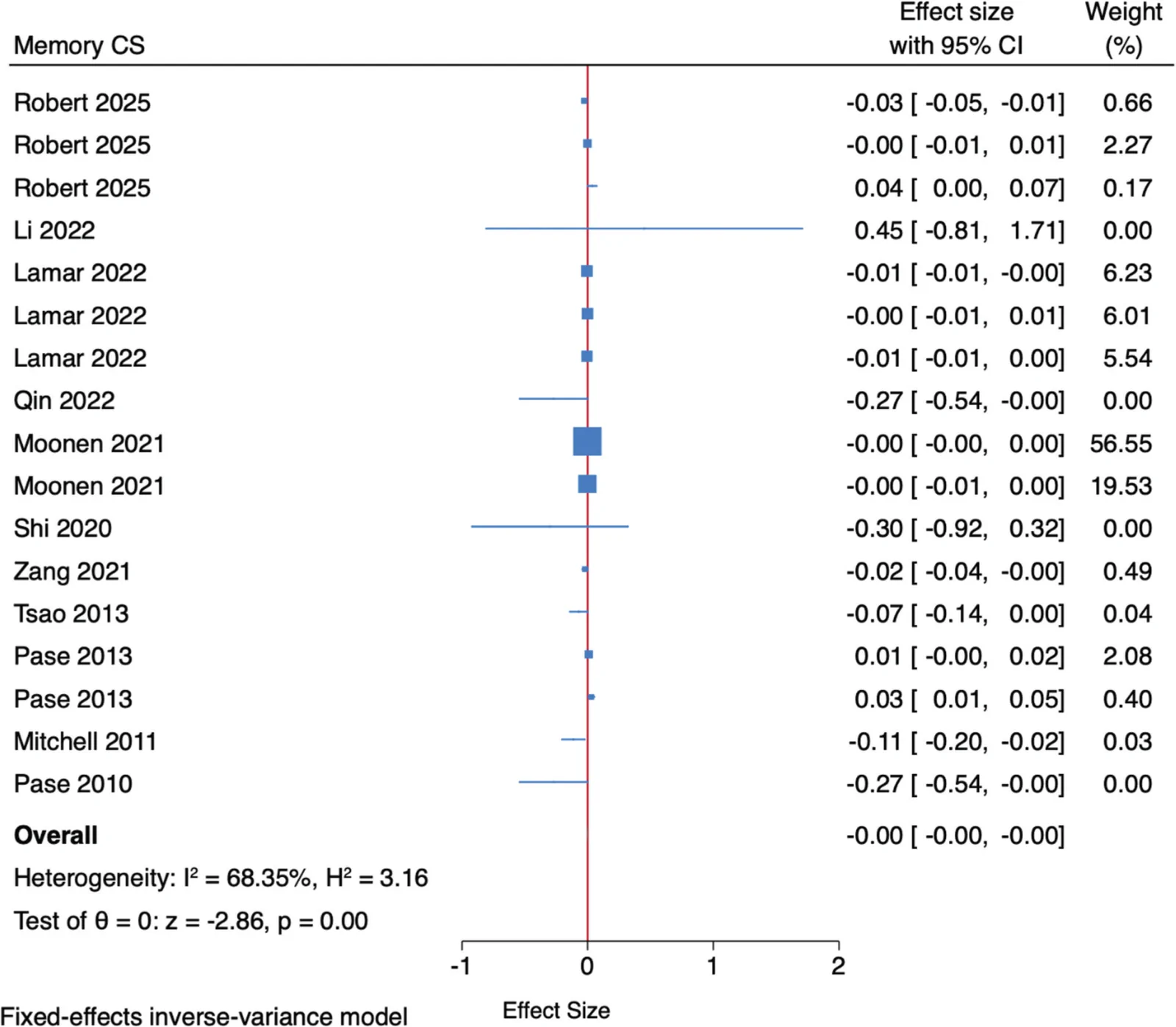
Meta-regression results for cross-sectional association of pulse pressure with memory.

Fifteen studies assessed the cross-sectional association between PP and memory (Table 1, Supplementary Table 4) involving 11 949 participants. Meta-analysis of 11 studies [15,33–42] reporting standardized beta coefficients or odds ratio converted to standardized beta-coefficients (Supplementary Table 5), gave a pooled standardized beta coefficient of –0.002 (95% CI: -0.004 to -0.001, *P* < 0.0042, Fig. 4) with evidence of heterogeneity between studies indicated by a wide range of standardized beta regression coefficient (-0.28 to 0.45 and *I*^2^ = 68.35%, *P* < 0.001). Publication bias was suggested from funnel plot asymmetry and Egger's test (*P* = 0.0101). All four studies that were not included in the meta-analysis, did not report any statistical findings [18,43].

**FIGURE 5:**
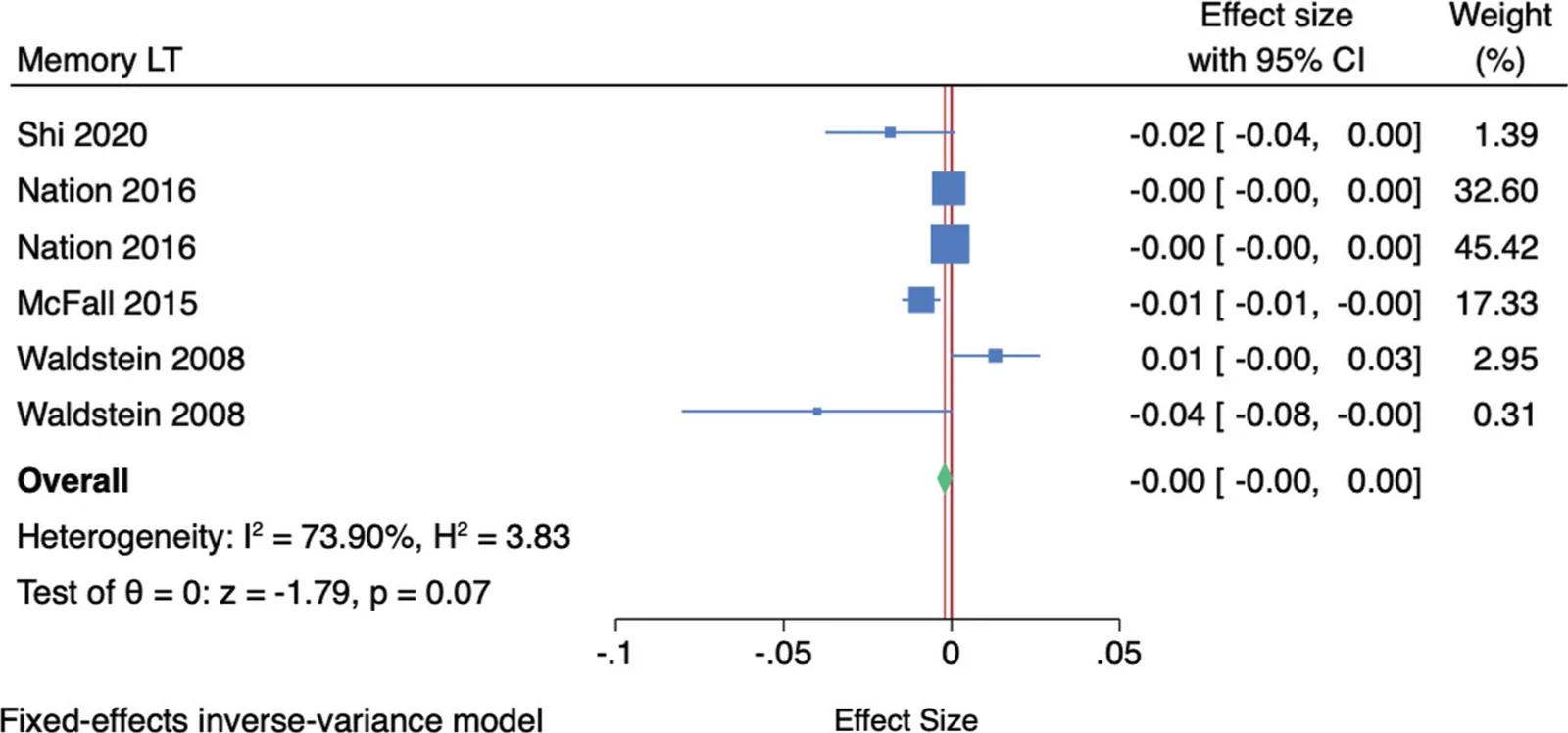
Meta-regression results for longitudinal association of pulse pressure with memory.

Four studies assessed the longitudinal association between PP and memory (Table 1) involving 5085 participants. Meta-analysis of four studies [29,41,44,45] reporting standardized beta coefficients or odds ratio converted to standardized beta-coefficients (Supplementary Table 6) gave a pooled standardized beta coefficient of –0.002 (95% CI: -0.004 to 0.001, *P* = 0.0736, Fig. 5) with evidence of heterogeneity between studies indicated by a wide range of standardized beta regression coefficient (-0.040 to 0.013 and *I*^2^ = 73.90%, *P* = 0.0018). Publication bias was not suggested from funnel plot asymmetry and Egger's test (*P* = 0.1415).

### Language

**FIGURE 6:**
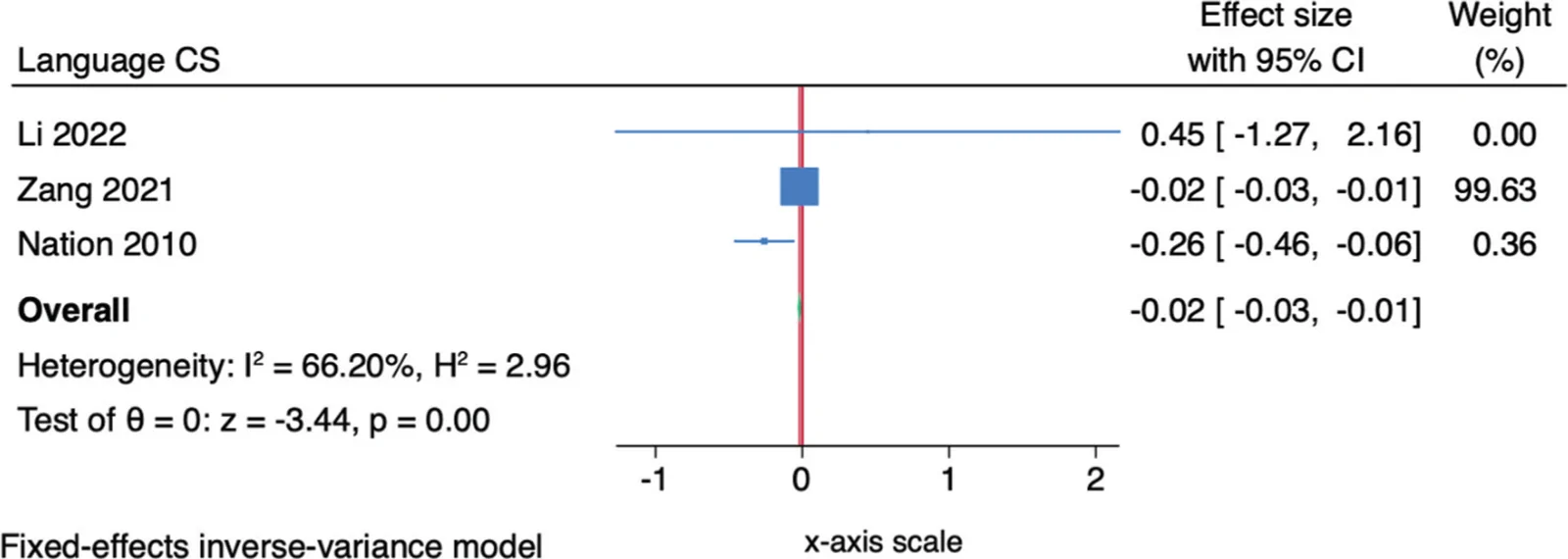
Meta-regression results for the cross-sectional association of pulse pressure with language.

Three studies assessed the cross-sectional association between PP and language (Table 1, Supplementary Table 7) involving 3308 participants. Meta-analysis of the three studies [15,22,34] reporting standardized beta-coefficient or odds ratio (converted to standardized beta-coefficient, Supplementary Table 8) gave a pooled standardized beta-coefficient of –0.021 (95% CI: -0.033 to –0.09, *P* < 0.001, Fig. 6) with evidence of heterogeneity between studies indicated by a wide range of standardized beta regression coefficient (-1.270 to 2.160 and *I*^2^ = 66.20%, *P* < 0.001). Publication bias was not suggested from funnel plot asymmetry and Egger's test (*P* = 0.7589).

Two studies assessed the longitudinal association between PP and language (Supplementary Table 7) involving 1677 participants. The studies reported inconsistent findings with one study showing no significant finding [45] and another reporting a significant negative association when language was assessed using the Similarities test [46].

### Attention

Four studies assessed the cross-sectional association between PP and attention (Supplementary Tables 9 and 10), involving 4007 participants. Two studies showed no significant association [18,34] and the remaining two studies found a negative association between PP and attention when assessed with MoCA Attention [15] and attention subscale of DRS [22].

**FIGURE 7:**
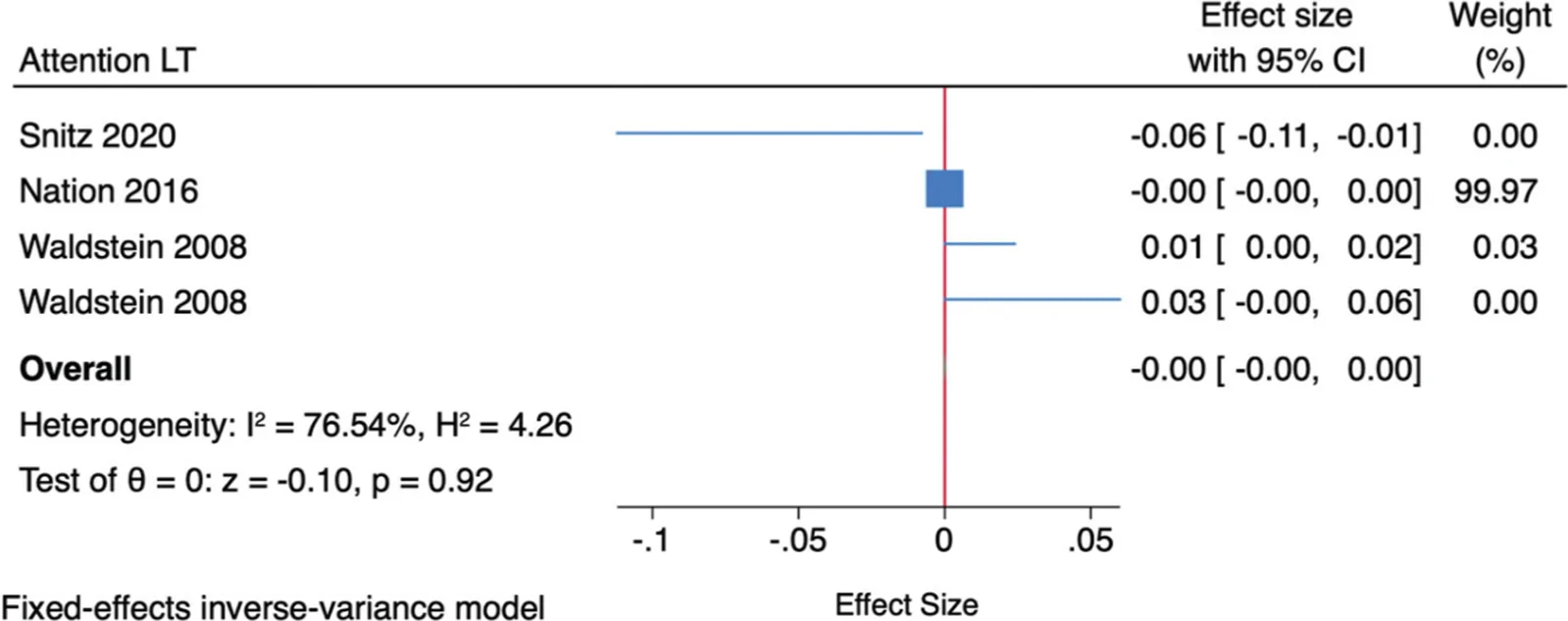
Meta-regression results for the longitudinal association of pulse pressure with attention.

Three studies assessed the longitudinal association between PP and attention (Supplementary Table 9) involving 1881 participants. Meta-analysis of the three studies [29,45,47] reporting standardized beta-coefficients or odds ratio (converted to standardized beta-coefficients, Supplementary Table 11) gave a pooled standardized beta coefficient of –0.001 (95% CI: -0.001 to 0.001, *P* = 0.9191, Fig. 7) with minimal evidence of heterogeneity between studies indicated by a small range of standardized beta regression coefficients (-0.06 to 0.03 and *I*^2^ = 76.54%, *P* = 0.05). Publication bias was not suggested from funnel plot asymmetry and Egger's test (*P* = 0.3541).

### Executive function

**FIGURE 8:**
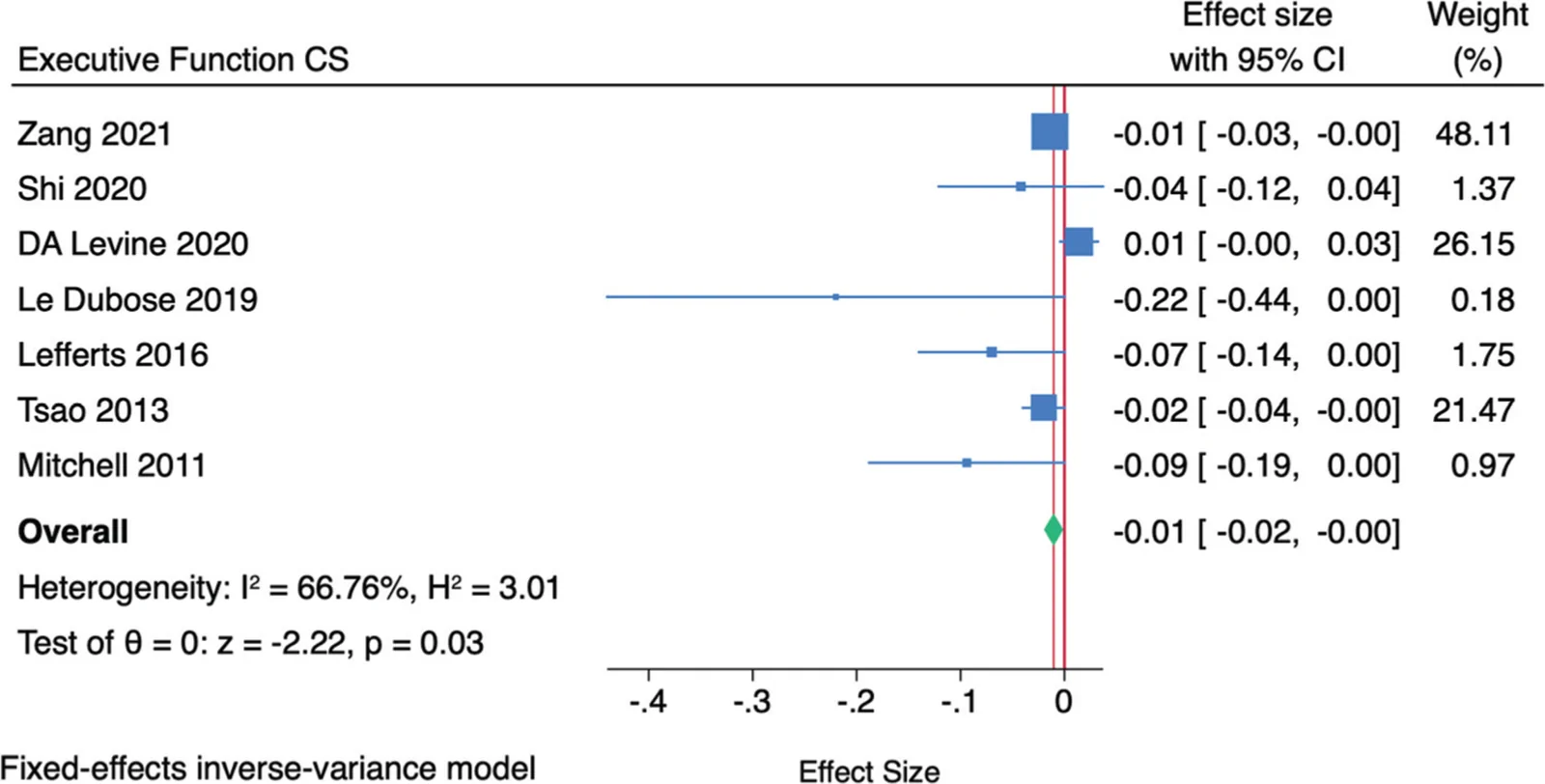
Meta-regression results for the cross-sectional association of pulse pressure with executive function.

Eleven studies assessed the cross-sectional association between PP and executive function (Supplementary Table 12) involving 10 312 participants. Meta-analysis of seven studies [12,15,35,41,42,48,49] reporting standardized beta coefficients or odds ratio (converted to standardized beta coefficients, Supplementary Table 13) gave a pooled standardized beta coefficient of –0.010 (95% CI: -0.020 to –0.001, *P* = 0.027, Fig. 8) with evidence of heterogeneity between studies indicated by a wide range of standardized beta regression coefficient (-0.220 to 0.014 and *I*^2^ = 66.76%, *P* = 0.006). Publication bias was suggested from funnel plot asymmetry and Egger's test (*P* = 0.0043). Of the four studies not included in the meta-analysis, three did not report any statistical association [18,43,50] with one study reporting a significant negative association between PP and executive function [22].

Longitudinally, seven studies assessed the association between PP and executive function (Supplementary Table 12) involving 7822 participants. Meta-analysis of five studies [29,41,45–47] reporting standardized beta coefficients or odds ratio (converted to standardized beta-coefficient, Supplement Table 14) gave a pooled standardized beta coefficient of –0.001 (95% CI, -0.001 to 0.001, *P* = 0.1830, Fig. 9) with evidence of heterogeneity between studies indicated by a wide range of standardized beta coefficients (-0.430 to 0.050 and *I*^2^ = 74.21%, *P* < 0.005). Publication bias was mot suggested from funnel plot asymmetry and Egger's test (*P* = 0.5846). Of the two studies not included in the meta-analysis, one did not find a significant association [51] when using Trails B, with the other not reporting any statistical findings [52].

**FIGURE 9:**
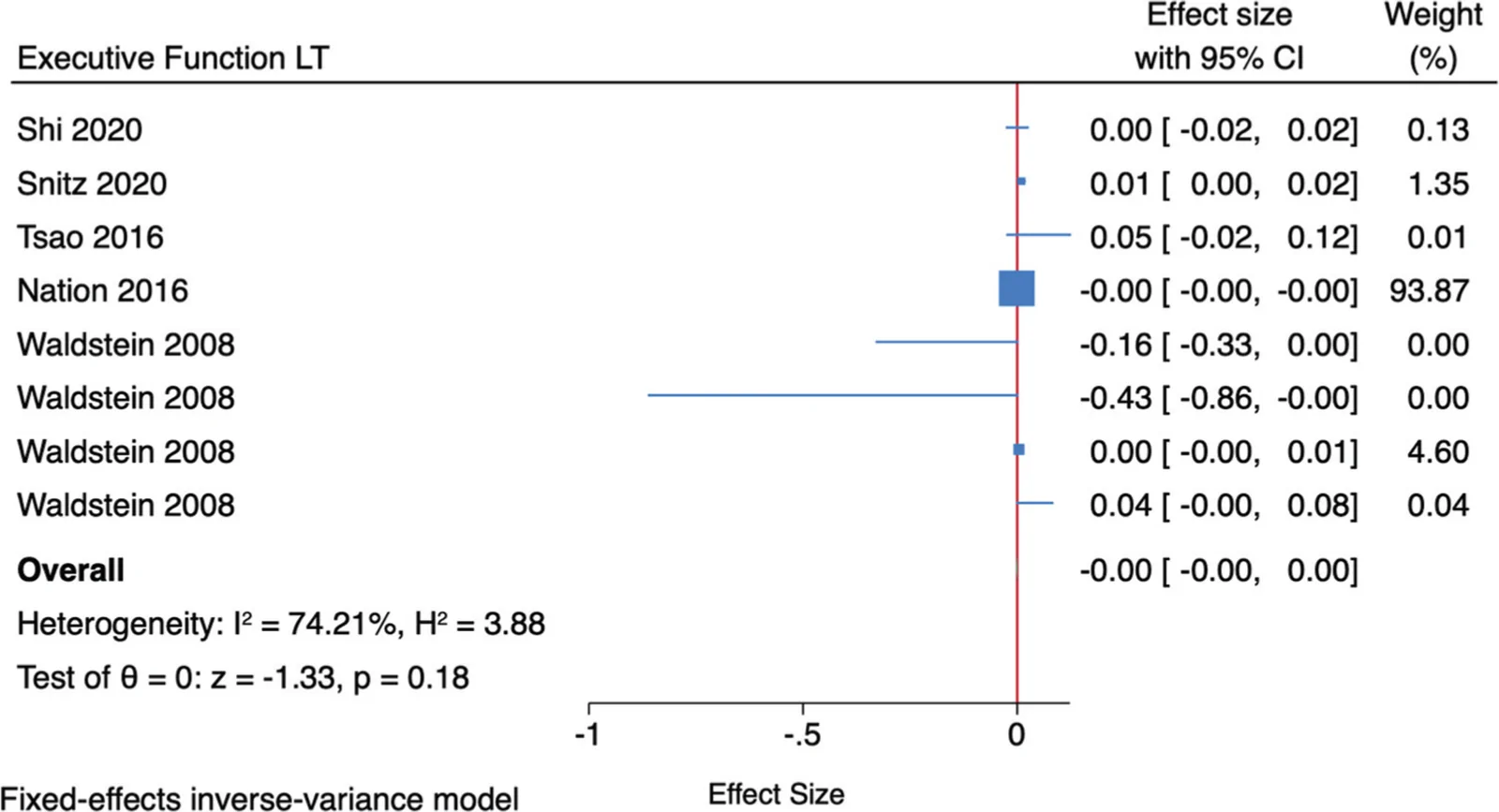
Meta-regression results for the longitudinal association of pulse pressure with executive function.

### Processing speed

**FIGURE 10:**
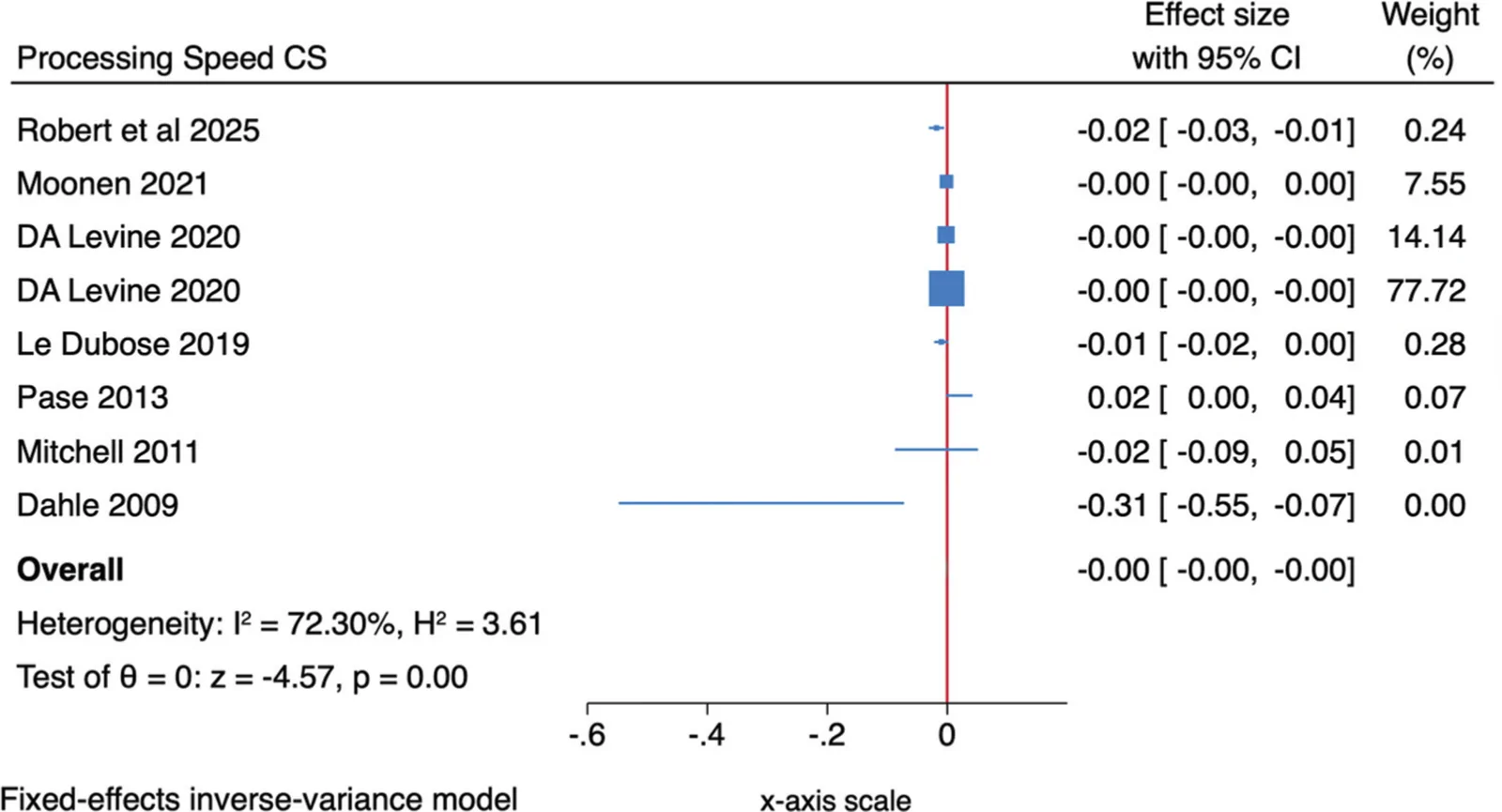
Meta-regression results for the cross-sectional association of pulse pressure with processing speed.

Eight studies assessed the cross-sectional association between PP and processing speed (Supplementary Table 15), involving 5937 participants. Meta-analysis of seven studies [12,35,36,38,40,43,48] reporting standardized beta coefficients or odds ratios (converted to standardized beta-coefficients, Supplementary Table 16), gave a pooled standardized beta coefficient of –0.001 (95% CI: -0.002 to –0.001, *P* < 0.001, Fig. 10), with evidence of heterogeneity between studies indicated by a wide range of standardized beta coefficients (-0.310 to 0.020 and *I*^2^ = 72.30%, *P* < 0.001). Publication bias was suggested from funnel plot asymmetry and Egger's test (*P* = 0.0059). The study not included in the meta-analysis [22] reported a significant negative association between PP and processing speed when assessing Trail Making Test Part A. No studies assessed the longitudinal association between PP and processing speed.

### Visuospatial ability

Two cross-sectional and one longitudinal study assessed the association between PP and visuospatial ability (Supplementary Table 17). Two studies did not report a significant association between PP and visuospatial ability when assessing line orientation and progressive matrices [33] or the Hooper Visual Organisation Test [45], with one study finding a significant negative association between PP and visuospatial ability when using the WISC and DSR assessment [22].

## DISCUSSION

The mechanisms linking cardiovascular disease and cognitive decline are incompletely understood. One hypothesis is that a chronic increase in pulsatile pressure flow reaching the brain causes damage to the brain microcirculation leading to cognitive decline [9]. Under normal conditions, cerebral blood flow autoregulation is maintained in a constant range. Higher pressure pulsatility passively dilates cerebral blood vessels favouring brain microvascular damage (microbleeds and ischemic attacks), white matter atrophy and grey matter thinning [9] which are hypothesized to lead to accelerated cognitive decline and dementia over time.

The aim of the present systematic review and meta-analysis was to investigate the association between PP and cognitive function assessed using several cognitive domains. We found evidence of a positive association between PP and global cognition and a negative association between PP and memory in cross-sectional and longitudinal studies. Processing speed, executive function and language negatively associated with PP in cross-sectional studies with limited evidence provided by longitudinal studies. There was limited evidence of an association with attention and visuospatial ability.

Global cognition is an individual's overall mental ability, assessing numerous cognitive domains such as memory, language and executive function, and is often assessed by tests such as MoCA and MMSE [53]. Brain regions linked with global cognition include the left amygdala, hippocampus, parietal lobule, superior temporal gyrus, insula and posterior temporal lobe [54]. The observed positive association between PP and global cognition from both cross-sectional and longitudinal studies is unclear and contrary to the present hypothesis. A possible explanation may be that higher pulsatile pressure may facilitate better brain perfusion as an adaptive mechanism. This is supported by the largest cross-sectional study and longitudinal studies by Lipnicki 2019 [17], a meta-analysis from 20-population based cohorts including 48 522 individuals, mean age of 72.7 without dementia at baseline. The longitudinal study had a 2 to 15-year follow-up, and overall, they found that no significant association with PP was found with global cognition or with cognitive decline. A mouse-model study by Winder *et al.* 2023 [55] in young and old mice found that there are age-related differences in the cerebral artery endothelial cell response to acute high PP. The study found that ex-vivo exposure to high PP impairs endothelium-dependent dilation in young cerebral arteries but does not affect old cerebral arteries. This suggests that higher stiffness of the old cerebral arteries is a possible mechanism for the protection against high PP-induced endothelial dysfunction. This is important as it suggests that cerebral arteries adapt to increases in pulse pressure with age, which may help to protect against declines in cognitive function.

Memory can be classified into three different systems based upon the type of information being processed and the time span it covers [56]. The three systems are: sensory/working memory, short-term memory and long-term memory. Numerous key brain structures are involved in different aspects of memory, with specific brain regions being involved in different types of memory [56]. Sensory/working memory is the briefest form of memory, lasting up to a few seconds, before the information is passed to short-term memory or letting it disappear. The temporal and occipital lobes are involved in sensory memory. Short-term/immediate memory is the retention of memory for up to 30 s. The hippocampal network, including the parahippocampal gyrus, hippocampus and neocortical areas, plays a key role in forming new memories and their subsequent reactivation [57]. The hippocampus is a complex brain structure embedded deep into the temporal lobe. It has been one of the most studied areas within the brain, and long-term potentiation was first discovered in this area [58]. The hippocampus is essential for the consolidation of information from short-term memories to long-term memories [56]. Although the hippocampus is crucial for forming long-term memories, other brain regions such as the basal ganglia, cerebellum and premotor cortex are some sites where memories are stored [59]. Episodic memory is a type of long-term memory that has the capacity to form and retrieve conscious memories of specific past events. Immediate and episodic memory remain relatively stable with age, while working memory can change significantly and impact daily activities. Tests included in the present systematic review used a variety of tests to assess different types of memory recall, for example, verbal, semantic and digit ordering, which mainly assess working and episodic memory. In the present systematic review, meta-analysis of both cross-sectional and longitudinal studies suggested a negative association between PP and memory.

We found evidence of a negative association between PP and processing speed in cross-sectional studies which is consistent with a previous systematic review looking at the association between blood pressure and hypertension and processing speed [60]. No studies assessed the longitudinal association between PP and processing speed. We found no evidence of an association between PP to attention or visuospatial ability; however, these two domains were the most underrepresented in the present systematic review with only four and two cross-sectional studies, respectively, investigating their association. Visuospatial memory remains relatively stable with age and may be relatively unaffected by stressors such as high PP. On the other hand, attention can vary significantly with age [61] and it has been suggested that lower levels of attention can increase pressure pulsatility [62].

Executive function in an umbrella term covering high-level cognitive processes and mental skills that allow individuals to plan and execute nonroutine behaviour to achieve their goals [63]. Although the brain mechanisms underlying executive function remain incompletely understood, brain regions implicated in executive function include the frontal lobe, particularly the prefrontal cortex, which are strongly interconnected with other subcortical structures such as the basal ganglia [64]. Tests designed to quantify executive function clinically (e.g. Wisconsin Card Sorting and Trail-Making tests) include switching between tasks, inhibiting strong impulses and sorting into categories [65]. However, executive function can be further sub-categorised into mental flexibility, verbal fluency, planning, working memory and inhibitory control, which are assessed to different degrees by neuropsychological tests. Evidence of a modest negative association between PP and executive function reported in the present systematic review is supported by cross-sectional with a pooled standardized regression coefficient of -0.01 (95% CI -0.02 to -0.00) with limited evidence from longitudinal studies. As executive function involves the coordination of several distributed cerebral function, it can be hypothesized that these interconnections rely on healthy white matter which is particularly prone to vascular injury and as such may explain the association between PP and executive function [66] but which may not be evidenced in longitudinal studies that include a short follow-up interval (5–7 year follow-up in largest study [45]). It is of note that the greatest white matter volume losses occur in the frontal lobe with age [67].

Cognitive performance is commonly assessed using cognitive function tests, however there is wide heterogeneity of cognitive screening tools and the cognitive domains that they assess. A single cognitive domain can be assessed using one or multiple tools, which varies between studies. Such variation adds considerable heterogeneity between studies. In addition, such tests are often subject to important individual limitations and vary in sensitivity and reproducibility. Furthermore, longitudinal use of cognitive function tests is subject to learning bias masking decline in cognitive function and favouring a false-negative finding [67]. Such variation may explain our findings of an inconsistent association between PP to language, attention, executive function, and visuospatial ability. Other possible sources of heterogeneity between studies, such as study design, populations included and the particular age groups and adjustment for different covariates, are also possible explanations for lack of consistency between studies. While language and some forms of memory (immediate and episodic) remain relatively stable with age; global cognition, working memory and attention can change significantly and impact daily activities significantly. As such, understanding mechanisms that may accelerate these changes is a key focus of dementia research.

The present systematic review benefits from including publications investigating the association between PP and cognitive function tests assessing different cognitive domains. However, several limitations require highlighting. Where possible, we performed a meta-regression analysis, pooling together studies to get an effect size estimate. For this analysis, reported summary data were used rather than using original data and where needed summary statistics were converted to beta-coefficient and standard errors estimated from CI which is likely to introduce some error in the pooled ES. From meta-regression analysis, there was evidence of heterogeneity in included studies. Therefore, these were corroborated with narratively summarized findings. There were no longitudinal studies assessing the association between PP and processing speed and thus we are unable to confirm cross-sectional associations.

In conclusion, we found limited evidence of an association between PP and cognitive function domains. The inconsistency in findings in the present systematic review is likely explained by heterogeneity in utilized cognitive tests, population studies, sample size and adjustment for covariates. The likely complex association between PP and cognitive function, which is likely to be dynamic and to change across the lifespan further. Large longitudinal studies, using standardized and well characterized cognitive tests, are needed to establish any association between PP and cognitive function.

## ACKNOWLEDGEMENTS

None.

Olivia Gaunt, Kaavya Venkatesh contributed equally and are co-first authors.

### Conflicts of interest

There are no conflicts of interest.

## Supplementary Material

Supplemental Digital Content
