# Supplementary material for: Association between pulse pressure and markers of cognitive function: a systematic review and meta-analysis
Source: J Hypertens. 2026 Jul 24;44(10):1692–707. doi: 10.1097/HJH.0000000000004401 (PMC13566397; doi:10.1097/HJH.0000000000004401)
Supplement: Supplementary file 1 [file jhype-44-1692-s001.docx]

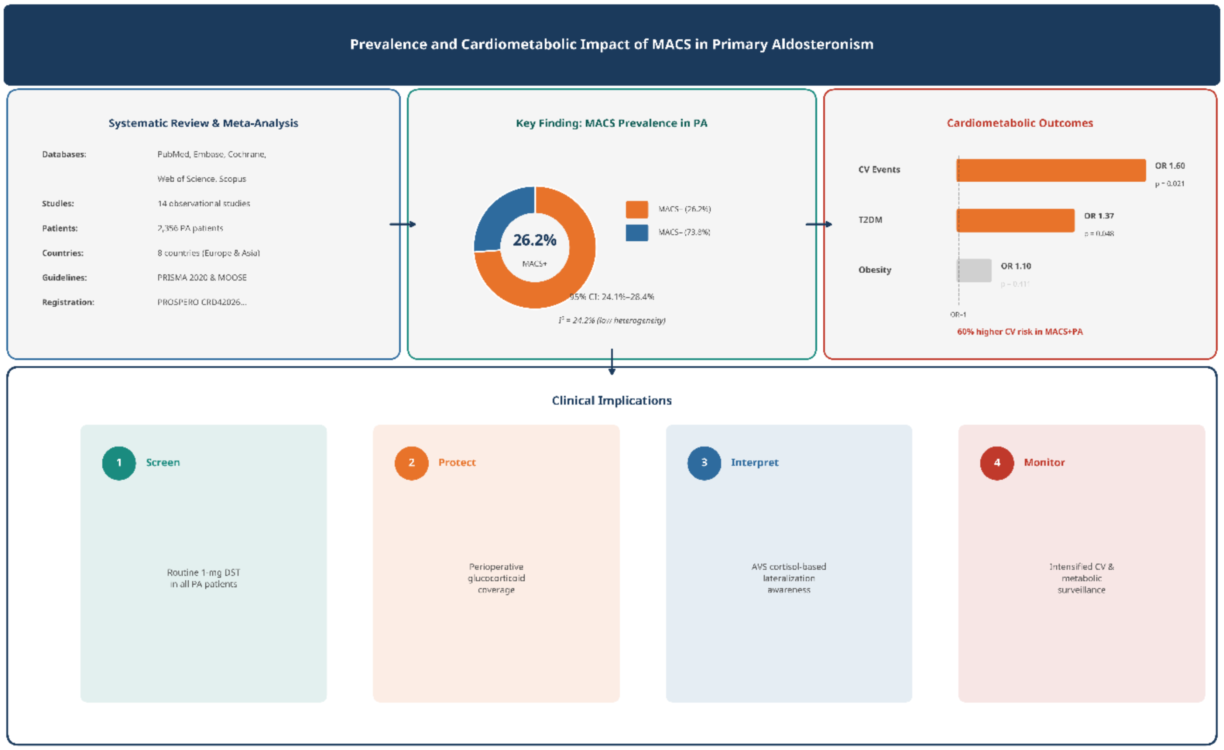

MACS = Mild Autonomous Cortisol Secretion | PA = Primary Aldosteronism | DST = Dexamethasone Suppression Test | AVS = Adrenal Venous Sampling
